# On the Consistency between a Classical Definition of the Geoid-to-Quasigeoid Separation and Helmert Orthometric Heights

**DOI:** 10.3390/s23115185

**Published:** 2023-05-30

**Authors:** Robert Tenzer, Albertini Nsiah Ababio

**Affiliations:** Department of Land Surveying and Geo-Informatics, Hong Kong Polytechnic University, Hong Kong; robert.tenzer@polyu.edu.hk

**Keywords:** gravity, levelling, heights, vertical geodetic control, gravity gradient

## Abstract

It is acknowledged that a classical definition of the geoid-to-quasigeoid separation as a function of the simple planar Bouguer gravity anomaly is compatible with Helmert’s definition of orthometric heights. According to Helmert, the mean actual gravity along the plumbline between the geoid and the topographic surface in the definition of orthometric height is computed approximately from the measured surface gravity by applying the Poincaré-Prey gravity reduction. This study provides theoretical proof and numerical evidence that this assumption is valid. We demonstrate that differences between the normal and (Helmert) orthometric corrections are equivalent to the geoid-to-quasigeoid separation differences computed for individual levelling segments. According to our theoretical estimates, maximum differences between these 2 quantities should be less than ±1 mm. By analogy, differences between the Molodensky normal and Helmert orthometric heights at levelling benchmarks should be equivalent to the geoid-to-quasigeoid separation computed from the Bouguer gravity data. Both theoretical findings are inspected numerically by using levelling and gravity data along selected closed levelling loops of the vertical control network in Hong Kong. Results show that values of the geoid-to-quasigeoid separation at levelling benchmarks differ less than ±0.1 mm from differences between the normal and orthometric corrections. Relatively large differences (slightly exceeding 2 mm) between values of the geoid-to-quasigeoid separation and differences between the normal and (Helmert) orthometric heights at levelling benchmarks are explained by errors in levelling measurements rather than by inconsistencies in computed values of the geoid-to-quasigeoid separation and (Helmert) orthometric correction.

## 1. Introduction

Orthometric and normal heights are the two most commonly used types of heights for a practical realization of geodetic vertical controls by physically establishing levelling benchmarks. Both types of heights are determined from precise levelling and gravity measurements (along levelling lines). Since the realization of levelling networks and their maintenance is extremely costly, geoid and quasigeoid models have been used in many countries around the world for a realization of geodetic vertical datums (either solely or together with a levelling network). Detailed geoid/quasigeoid models are also indispensable for the conversion of geodetic (geometric) heights measured by the Global Navigation Satellite Systems (GNSS) techniques (such as GPS) for officially adopted orthometric/normal heights. In essence, concepts of defining orthometric and normal heights (or equivalently geoid or quasigeoid heights) depend on a treatment of the topographic density. Whereas the topographic density is taken into consideration in the definition of orthometric heights, Molodensky [1,2] completely disregarded the topographic density in the definition of normal heights (see also [3]). Normal heights can then directly be determined from levelling and gravity measurements. His main argument was that knowledge of the actual topographic density distribution will always be limited to the extent that the determination of orthometric heights with the same accuracy as could be achieved for normal heights cannot be guaranteed. 

Despite the fact that Molodensky’s argument was widely accepted by the geodetic community, the requirement of taking into consideration the topographic density in the definition of physical heights has also not been completely dismissed. The most obvious way of addressing a lack of knowledge about the actual topographic density distribution is to adopt a constant density value, preferably an average topographic density for a particular region where a levelling network is established. Following this principle, Helmert [4,5] defined orthometric heights so that the Poincaré-Prey gravity reduction is used to compute the mean gravity along the plumbline between the geoid and the topographic surface (i.e., within the topography) from the surface gravity value. According to his definition, orthometric heights are defined for a constant topographic density, but a terrain relief and the effect of a mass density distribution below the geoid on the actual gravity gradient are disregarded. Intuitively, the accuracy of Helmert orthometric heights could further be improved by taking into consideration terrain geometry. Such theoretical attempts (together with numerical examples) have been conducted by [6,7,8,9,10,11,12,13] and others (see also [14]). Tenzer and Vaníček [15] investigated the effect of (lateral) topographic density variations on orthometric heights. More advanced methods of computing the mean gravity value in the definition of orthometric heights have been proposed and numerically inspected by a number of authors, e.g., [16,17,18,19,20,21,22,23,24,25,26,27,28,29,30,31]. 

Until now, Helmert’s definition of orthometric heights was almost exclusively used in countries where this type of height is adopted, despite numerous studies addressing a possible improvement of the accuracy by reducing errors due to disregarding a topographic density distribution, terrain relief, and density heterogeneities below the geoid surface. The Molodensky normal and Helmert orthometric heights are, thus, the most commonly used types of heights for a practical realization of geodetic vertical datums around the world. The difference between these two types of heights (i.e., the geoid-to-quasigeoid separation) is defined by means of applying the Poincaré-Prey gravity gradient to approximate the actual gravity gradient in the computation of the mean gravity value along the plumbline within the topography. This formulation yields the expression for computing the geoid-to-quasigeoid separation from the simple (incomplete) planar Bouguer gravity anomaly and the height of the computation point e.g., [32] and Equations (8)–(103). This expression is very often used for the conversion of Molodensky normal to Helmert orthometric heights (and vice versa) as well as between the geoid and quasigeoid models. Again, more refined numerical models have been developed and applied to define the geoid-to-quasigeoid separation, e.g., [28,31,33,34,35,36,37,38,39,40,41,42,43,44,45,46,47], in terms of differences between the geoid and quasigeoid heights, both defined by means of the Bruns’ theorem [48]; however, their practical applications are still rare. 

As stated above, the computation of the geoid-to-quasigeoid separation from the Bouguer gravity data should provide results that agree with differences between the Helmert orthometric and Molodensky normal heights, because the same assumptions were adopted in both definitions. This study investigates this aspect by providing theoretical proof that both definitions are fully compatible, except for small errors due to adopted approximations that are completely negligible. Theoretical findings are then numerically inspected by using levelling and gravity data. The study is organized into five sections. Theoretical definitions are briefly reviewed in Section 2. The methodology applied in numerical studies is explained in Section 3. Results are presented and discussed in Section 4 and Section 5, respectively, and the study is concluded in Section 6. 

## 2. Theory

This section briefly summarizes definitions of heights, the geoid-to-quasigeoid separation, and the normal and orthometric corrections. Rigorous (i.e., accurate) and approximate expressions for the geoid-to-quasigeoid separation difference and its relationship with the normal and orthometric correction difference for a levelling segment are derived. 

### 2.1. Normal and Orthometric Heights

**Figure 1:**
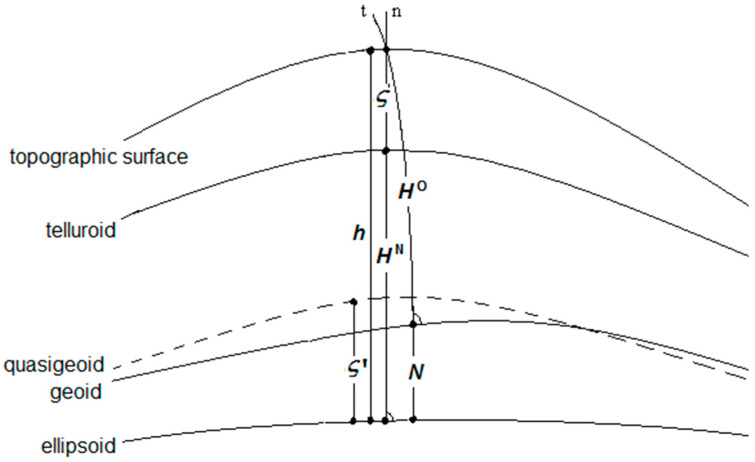
Height systems: the normal height $H^{N}$, the orthometric height $H^{O}$, the geodetic (ellipoidal) height h, the geoid height N, the height anomaly ς, and the quasigeoid height $\varsigma^{\prime }$. The plumbline and ellipsoidal normal are denoted as t and n, respectively.

The orthometric height $H^{O}$ is defined by (e.g., [32] and Equations (4)–(21))

(1) $$H^{O}=\frac{C}{\bar{g}}$$

where C is the geopotential number of a point at the topographic surface, and $\bar{g}$ is the mean actual gravity along the plumbline within the topography (see Figure 1).

The normal height $H^{N}$ was defined by [1,2] in the following form

(2) $$H^{N}=\frac{C}{\bar{\gamma }}$$

where the mean normal gravity $\bar{\gamma }$ along the ellipsoidal normal between the reference ellipsoid and the telluroid (i.e., the surface on which the normal potential is equal to the actual potential at the topographic surface) is evaluated according to the Somigliana–Pizzetti’s theory of the normal gravity field [49,50]. 

The geopotential number C in definitions of orthometric and normal heights (Equations (1) and (2)) is practically computed from measured levelling height differences $\Delta H_{i}$ and observed gravity values $g_{i}$ along levelling lines, i.e., $C=\sum_{i}g_{i}\Delta H_{i}$. When normal gravity values are used instead of observed gravity values along levelling lines, the vertical geodetic datum is realized in the system of normal-orthometric heights $H^{N-O}$ defined by (e.g., [17])

(3) $$H^{N-O}=\frac{C^{N}}{\bar{\gamma }}$$

where the normal geopotential number $C^{N}$ is computed from measured levelling height differences $\Delta H_{i}$ and normal gravity values $\gamma_{i}$ along levelling lines, i.e., $C^{N}=\sum_{i}\gamma_{i}\Delta H_{i}$. 

Heights of levelling benchmarks in some countries and territories (such as Hong Kong) have been determined only from levelling measurements, involving neither the actual nor normal gravity information. In this case, heights H of levelling benchmarks are directly computed from measured levelling height differences $\Delta H_{i}$ so that

(4) $$H=\sum_{i}\Delta H_{i}$$

### 2.2. Difference between the Normal and Orthometric Corrections

Heights H obtained from levelling measurements (Equation (4)) are typically converted to either orthometric heights $H^{O}$ (Equation (1)) or normal heights $H^{N}$ (Equation (2)) by applying the orthometric or normal corrections to levelled height differences $\Delta H_{i}$, respectively. It is worth noting that heights H could eventually be first converted to normal-orthometric heights (Equation (3)) by using normal gravity values computed along levelling lines, and consequently to the normal heights by applying the cumulative normal to normal-orthometric height correction [51,52,53]. This two-step numerical scheme is obviously not beneficial if we are not particularly interested in determining this type of height. 

The orthometric correction $OC_{i,i+1}$ of a levelling segment between two benchmarks *i* and *i* + 1 is defined by (e.g., [32] (Equations (4)–(33))

(5) $$OC_{i,i+1}=\sum_{k=i}^{i+1}\frac{g_{k}-\gamma_{0}}{\gamma_{0}}\;\delta H_{k}+\frac{{\bar{g}}_{i}-\gamma_{0}}{\gamma_{0}}H_{i}-\frac{{\bar{g}}_{i+1}-\gamma_{0}}{\gamma_{0}}H_{i+1}$$

where $H_{i}$ and $H_{i+1}$ are heights of levelling benchmarks *i* and *i* + 1, respectively, ${\bar{g}}_{i}$ and ${\bar{g}}_{i+1}$ are the corresponding mean gravity values (as defined in Equation (1)), and $\delta H_{k}$ are levelled height differences (at levelling setups *k* between two benchmarks *i* and *i* + 1 of a levelling segment, i.e., $\Delta H_{i,i+1}=\sum_{k=i}^{i+1}\delta H_{k}$). The normal gravity $\gamma_{0}$ at the reference ellipsoid in Equation (5) is a constant value, meaning that it is computed for the same geodetic latitude, for instance φ=45°. 

The normal correction $NC_{i,i+1}$ is given by (e.g., [32] (Equations (4)–(45))

(6) $$NC_{i,i+1}=\sum_{k=i}^{i+1}\frac{g_{k}-\gamma_{0}}{\gamma_{0}}\;\delta H_{k}+\frac{{\bar{\gamma }}_{i}-\gamma_{0}}{\gamma_{0}}H_{i}-\frac{{\bar{\gamma }}_{i+1}-\gamma_{0}}{\gamma_{0}}H_{i+1}$$

where ${\bar{\gamma }}_{i}$ and ${\bar{\gamma }}_{i+1}$ are the mean normal gravity values (as defined in Equation (2)). As seen from the comparison of Equations (5) and (6), the mean normal gravity values ${\bar{\gamma }}_{i}$ and ${\bar{\gamma }}_{i+1}$ are used in the definition of the normal correction $NC_{i,i+1}$ instead of the mean gravity values ${\bar{g}}_{i}$ and ${\bar{g}}_{i+1}$ in the orthometric correction $OC_{i,i+1}$.

From Equations (5) and (6), the normal and orthometric correction difference (i.e., the difference between the normal and orthometric corrections) of an individual levelling segment between two benchmarks *i* and *i* + 1 is found to be

(7) $$\begin{array}{l} NC_{i,i+1}-OC_{i,i+1}=\sum_{k=i}^{i+1}\frac{g_{k}-\gamma_{0}}{\gamma_{0}}\;\delta H_{k}-\sum_{k=i}^{i+1}\frac{g_{k}-\gamma_{0}}{\gamma_{0}}\;\delta H_{k}\\ +\frac{{\bar{\gamma }}_{i}-\gamma_{0}}{\gamma_{0}}H_{i}-\frac{{\bar{g}}_{i}-\gamma_{0}}{\gamma_{0}}H_{i}-\frac{{\bar{\gamma }}_{i+1}-\gamma_{0}}{\gamma_{0}}H_{i+1}+\frac{{\bar{g}}_{i+1}-\gamma_{0}}{\gamma_{0}}H_{i+1}\\ =\frac{H_{i}}{\gamma_{0}}\left({\bar{\gamma }}_{i}-\gamma_{0}-{\bar{g}}_{i}+\gamma_{0}\right)+\frac{H_{i+1}}{\gamma_{0}}\left({\bar{g}}_{i+1}-\gamma_{0}-{\bar{\gamma }}_{i+1}+\gamma_{0}\right)\\ =\frac{H_{i}}{\gamma_{0}}\left({\bar{\gamma }}_{i}-{\bar{g}}_{i}\right)-\frac{H_{i+1}}{\gamma_{0}}\left({\bar{\gamma }}_{i+1}-{\bar{g}}_{i+1}\right)\\ =\frac{H_{i+1}}{\gamma_{0}}\left({\bar{g}}_{i+1}-{\bar{\gamma }}_{i+1}\right)-\frac{H_{i}}{\gamma_{0}}\left({\bar{g}}_{i}-{\bar{\gamma }}_{i}\right) \end{array}$$

Equation (7) defines the normal and orthometric correction difference rigorously as a function of heights $H_{i}$ and $H_{i+1}$ of benchmarks determined from levelling measurements. In addition, the functional relation involves differences between the mean actual and normal gravity values ${\bar{g}}_{i}-{\bar{\gamma }}_{i}$ and ${\bar{g}}_{i+1}-{\bar{\gamma }}_{i+1}$ (at locations of benchmarks *i* and *i* + 1). 

### 2.3. Geoid-to-Quasigeoid Separation Difference 

The geoid-to-quasigeoid separation χ can be defined as a difference between the normal and orthometric heights so that

(8) $$\chi =H^{N}-H^{O}$$

By analogy with Equation (8), the geoid-to-quasigeoid separation difference $\Delta \chi_{i,i+1}$ for a levelling segment (between two benchmarks *i* and *i* + 1) can be defined in the following form:

(9) $$\Delta \chi_{i,i+1}=\chi_{i+1}-\chi_{i}=\left(H_{i+1}^{N}-H_{i+1}^{O}\right)-\left(H_{i}^{N}-H_{i}^{O}\right)$$

The orthometric height difference $\Delta H_{i,i+1}^{O}$ (between two levelling benchmarks *i* and *i* + 1) is obtained by applying the orthometric correction $OC_{i,i+1}$ to the levelled height difference $\Delta H_{i,i+1}$ so that

(10) $$H_{i+1}^{O}-H_{i}^{O}=\Delta H_{i,i+1}^{O}=\Delta H_{i,i+1}+OC_{i,i+1}$$

The application of the normal correction $NC_{i,i+1}$ to the levelled height difference $\Delta H_{i,i+1}$ yields the normal height difference $\Delta H_{i,i+1}^{N}$. Hence,

(11) $$H_{i+1}^{N}-H_{i}^{N}=\Delta H_{i,i+1}^{N}=\Delta H_{i,i+1}+NC_{i,i+1}$$

By combining Equations (9)–(11), the following relation is found:

(12) $$\Delta H_{i,i+1}^{N}-\Delta H_{i,i+1}^{O}=\left(H_{i+1}^{N}-H_{i+1}^{O}\right)-\left(H_{i}^{N}-H_{i}^{O}\right)\;\;=\left(H_{i+1}^{N}-H_{i}^{N}\right)-\left(H_{i+1}^{O}-H_{i}^{O}\right)\;\;=\Delta H_{i,i+1}+NC_{i,i+1}-\Delta H_{i,i+1}-OC_{i,i+1}\;\;=NC_{i,i+1}-OC_{i,i+1}$$

As seen in Equation (12), the difference between the normal and orthometric height differences $\Delta H_{i,i+1}^{N}-\Delta H_{i,i+1}^{O}$ directly equals the difference between the normal and orthometric corrections $NC_{i,i+1}-OC_{i,i+1}$. 

Substitution from Equation (7) to Equation (12) yields

(13) $$\Delta H_{i,i+1}^{N}-\Delta H_{i,i+1}^{O}=NC_{i,i+1}-OC_{i,i+1}=\frac{H_{i+1}}{\gamma_{0}}\left({\bar{g}}_{i+1}-{\bar{\gamma }}_{i+1}\right)-\frac{H_{i}}{\gamma_{0}}\left({\bar{g}}_{i}-{\bar{\gamma }}_{i}\right)$$

By combining Equations (9) and (13), the following expression is obtained:

(14) $$\Delta \chi_{i,i+1}=\chi_{i+1}-\chi_{i}=NC_{i,i+1}-OC_{i,i+1}=\frac{H_{i+1}}{\gamma_{0}}\left({\bar{g}}_{i+1}-{\bar{\gamma }}_{i+1}\right)-\frac{H_{i}}{\gamma_{0}}\left({\bar{g}}_{i}-{\bar{\gamma }}_{i}\right)$$

Equation (14) defines the geoid-to-quasigeoid separation difference $\Delta \chi_{i,i+1}$ for a levelling segment (between two benchmarks *i* and *i* + 1) in terms of the normal and orthometric correction difference $NC_{i,i+1}-OC_{i,i+1}$ that was introduced in Equation (7). 

### 2.4. Approximate Definition of the Geoid-to-Quasigeoid Separation 

As stated in Section 1, orthometric heights in all countries are defined according to Helmert’s theory [4,5]. By analogy, the geoid-to-quasigeoid separation $\chi^{\prime }$ is defined as a difference between the Molodensky normal height $H^{N}$ and the Helmert orthometric height ${\tilde{H}}^{O}$. Despite this definition being well known and readily found in geodetic literature, e.g., [32], it is worth recapitulating derivations of its approximate form $\chi^{\prime }\;=H^{N}-{\tilde{H}}^{O}$ from its rigorous definition $\chi =H^{N}-H^{O}$ (given in Equation (8)) in order to better understand the adopted assumptions concerning the accuracy. 

Inserting from Equations (1) and (2) to Equation (8), the geoid-to-quasigeoid separation χ becomes

(15) $$\chi =H^{N}-H^{O}=\frac{C}{\bar{\gamma }}-\frac{C}{\bar{g}}=\frac{C}{\bar{\gamma }\bar{g}}\left(\bar{g}-\bar{\gamma }\right)=\frac{H^{O}}{\bar{\gamma }}\left(\bar{g}-\bar{\gamma }\right)$$

As seen in Equation (15), the geoid-to-quasigeoid separation is defined rigorously as a function of the difference between the mean actual and normal gravity values. The mean normal gravity $\bar{\gamma }$ in the denominator of Equation (15) can be replaced by the normal gravity value $\gamma_{0}$ at the reference ellipsoid. If the mean normal gravity $\bar{\gamma }$ is defined as a function of the normal gravity value $\gamma_{0}$ and the normal (linear) gravity gradient ∂γ/∂h, i.e., $\bar{\gamma }≅\gamma_{0}+\partial \gamma /\partial h\;\left(H/2\right)$, the difference between using $\gamma_{0}$ instead of $\bar{\gamma }$ introduces the following approximation error:

(16) $$\varepsilon_{\bar{\gamma }-\gamma_{0}}=\bar{\gamma }-\gamma_{0}≅\gamma_{0}+\frac{\partial \gamma }{\partial h}\frac{H}{2}-\gamma_{0}=\frac{\partial \gamma }{\partial h}\frac{H}{2}\approx -2\frac{\mathrm{GM}}{R^{3}}\frac{H}{2}=-\frac{\mathrm{GM}}{R^{3}}H$$

where $\gamma ≅{\mathrm{GM}/R}^{2}$ and, consequently, $\partial \gamma /\partial h\;≅-2{\mathrm{GM}/R}^{3}$ are defined in terms of the Earth’s mean radius R = 6371 × 10^3^ m and the geocentric gravitational constant GM = 3.986 × 10^14^ m^3^ s^−2^ (i.e., the product of the Newton gravitational constant G and the total mass of the Earth M). For maximum elevations of H≈ 9 km (in the Himalayas), the approximation error could reach maximum of $\varepsilon_{\bar{\gamma }-\gamma_{0}}\approx$ 0.014 m s^−2^ (Equation (16)). In Hong Kong, these errors are obviously much smaller. 

The error $\varepsilon_{\bar{\gamma }-\gamma_{0}}$ propagates into the error in values of the geoid-to-quasigeoid separation $\varepsilon_{\chi_{\gamma }}$ as follows:

(17) $$\varepsilon_{\chi_{\gamma }}=\frac{H^{O}}{\bar{\gamma }}\left(\bar{g}-\bar{\gamma }\right)-\frac{H^{O}}{\gamma_{0}}\left(\bar{g}-\bar{\gamma }\right)\;\;=\frac{H^{O}}{\bar{\gamma }\gamma_{0}}\left(\bar{g}-\bar{\gamma }\right)\left(\gamma_{0}-\bar{\gamma }\right)=\chi \frac{\left(\gamma_{0}-\bar{\gamma }\right)}{\gamma_{0}}=-\chi \frac{\varepsilon_{\bar{\gamma }-\gamma_{0}}}{\gamma_{0}}$$

Note that a standard error propagation was not applied to derive the expression in Equation (17), because only the mean normal gravity $\bar{\gamma }$ in the denominator of Equation (15) is approximated. Moreover, nonlinear gravity changes are disregarded, having no impact on the error analysis.

Substitution from Equation (16) to Equation (17) yields

(18) $$\varepsilon_{\chi_{\gamma }}≅\chi \frac{R^{2}}{\mathrm{GM}}\frac{\mathrm{GM}}{R^{3}}H=\chi \frac{H}{R}$$

For maximum values of the geoid-to-quasigeoid separation χ within ±5 m, the error $\varepsilon_{\chi_{\gamma }}$ is less than ±7 mm in mountainous regions with extreme elevations (particularly in the Himalayas, Tibet, and the Andes). Elsewhere, this error is typically less than ±1 mm. The geoid-to-quasigeoid separation χ in Equation (15) can then be defined as follows:

(19) $$\chi =H^{N}-H^{O}=\frac{H^{O}}{\bar{\gamma }}\left(\bar{g}-\bar{\gamma }\right)≅\frac{H^{O}}{\gamma_{0}}\left(\bar{g}-\bar{\gamma }\right)$$

Approximations adopted in Helmert’s definition of orthometric heights are applied to the difference between the mean actual and normal gravity values $\bar{g}-\bar{\gamma }$ in Equation (19). As already mentioned in Equation (16), the mean normal gravity $\bar{\gamma }$ is described as follows:

(20) $$\bar{\gamma }\approx \gamma_{0}+\frac{\partial \gamma }{\partial h}\frac{H^{N}}{2}$$

Note that in Equation (20), the normal height $H^{N}$ is used in the definition of the mean normal gravity according to Molodensky [1,2,3]. 

The mean actual gravity gradient in Equation (19) is further approximated the Poincaré-Prey gravity gradient so that

(21) $$\bar{g}≅g-\frac{\partial g}{\partial H}\frac{H^{O}}{2}\approx g-\left(\frac{\partial \gamma }{\partial h}+4\;\pi \;G\;\rho^{T}\right)\;\frac{H^{O}}{2}\;\approx g-\frac{\partial \gamma }{\partial h}\frac{H^{O}}{2}-2\;\pi \;G\;\rho^{T}H^{O}$$

The actual mean gravity in Equation (21) is first described as a function of the surface gravity g and the actual gravity gradient ∂g/∂H. The actual gravity gradient is then approximated by the Poincaré-Prey gravity gradient, $\partial g/\partial H\approx \partial \gamma /\partial h+4\;\pi \;G\;\rho^{T}$, which is defined as the normal linear gravity gradient ∂γ/∂h and the term $4\;\pi \;G\;\rho^{T}$ (i.e., the Poisson equation). An average upper continental crustal density of 2670 kg m^−3^ [54] is typically adopted as a topographic density ρ^T^ in geodetic and geophysical applications. It is worth noting that the actual topographic density could vary substantially with respect to the average topographic density of 2670 kg m^−3^. In the Hong Kong territories, for instance, the igneous and sedimentary rocks of lower densities represent most of the geological setting. Nevertheless, the density value of 2670 kg m^−3^ is, until now, exclusively used to define the (Helmert) orthometric heights in countries around the world where this type of height is adopted officially for a realization of the geodetic vertical control. Consequently, this density value is used to compute the geoid-to-quasigeoid separation. 

Combining Equations (20) and (21), the following expression is found:

(22) $$\bar{g}-\bar{\gamma }≅g-\frac{\partial \gamma }{\partial h}\frac{H^{O}}{2}-2\;\pi \;G\;\rho^{T}H^{O}-\gamma_{0}-\frac{\partial \gamma }{\partial h}\frac{H^{N}}{2}$$

Assuming that

(23) $$\frac{\partial \gamma }{\partial h}\frac{H^{O}}{2}+\frac{\partial \gamma }{\partial h}\frac{H^{N}\left(\Omega \right)}{2}≅\frac{1}{2}\frac{\partial \gamma }{\partial h}\left(H^{O}+H^{N}\right)≅\frac{\partial \gamma }{\partial h}H^{O}$$

Equation (22) further simplifies to

(24) $$\bar{g}-\bar{\gamma }≅g-\gamma_{0}-2\;\pi \;G\;\rho^{T}H^{O}-\frac{\partial \gamma }{\partial h}H^{O}$$

Note that approximations applied in Equations (22)–(24) do not affect the accuracy. Inserting from Equation (24) to Equation (19), the geoid-to-quasigeoid separation is then obtained in the following form:

(25) $$H^{N}-H^{O}≅\frac{H^{N}}{\gamma_{0}}\left(g-\gamma_{0}-\frac{\partial \gamma }{\partial h}H^{O}-2\;\pi \;G\;\rho^{T}H^{O}\right)$$

From a definition of the free-air gravity anomaly $\Delta g^{\mathrm{FA}}$, i.e.,

(26) $$\Delta g^{\mathrm{FA}}=g-\gamma_{0}-\frac{\partial \gamma }{\partial h}H^{O}$$

the expression in Equation (25) becomes

(27) $$H^{N}-H^{O}≅\frac{H^{N}}{\gamma_{0}}\left(\Delta g^{\mathrm{FA}}-2\;\pi \;G\;\rho^{T}H^{O}\right)$$

The simple planar Bouguer gravity anomaly $\Delta g^{\mathrm{SPB}}$ is computed from the free-air gravity anomaly $\Delta g^{\mathrm{FA}}$ by applying the Bouguer gravity reduction. Hence,

(28) $$\Delta g^{\mathrm{SPB}}=\Delta g^{\mathrm{FA}}-2\;\pi \;G\;\rho^{T}H^{O}$$

Substitution from Equation (28) to Equation (27) yields (e.g., [32] (Equations (8)–(103))

(29) $$\chi^{\prime }≅\frac{H^{N}}{\gamma_{0}}\Delta g^{\mathrm{SPB}}≅\frac{H}{\gamma_{0}}\Delta g^{\mathrm{SPB}}$$

Equation (29) defines the geoid-to-quasigeoid separation $\chi^{\prime }$ approximately as a function of the simple planar Bouguer gravity anomaly $\Delta g^{\mathrm{SPB}}$ and the normal height $H^{N}$. Note that heights H (obtained from levelling measurements) can be considered instead of $H^{N}$ and $H^{O}$ in Equations (29) and (28) without affecting the accuracy of $\chi^{\prime }$. 

### 2.5. Approximate Definition of the Geoid-to-Quasigeoid Separation Difference 

In Equation (14), the relation between the geoid-to-quasigeoid separation difference and the normal and orthometric correction difference (for a levelling segment between two benchmarks *i* and *i* + 1) was rigorously formulated. To find the corresponding approximate definition of the geoid-to-quasigeoid separation difference, the rigorous definition of the geoid-to-quasigeoid separation was first used to derive the rigorous definition of the geoid-to-quasigeoid separation difference. From Equation (15), we can write

(30) $$\Delta \chi_{i,i+1}=\chi_{i+1}-\chi_{i}=\frac{H_{i+1}^{O}}{\gamma_{0}}\left({\bar{g}}_{i+1}-{\bar{\gamma }}_{i+1}\right)-\frac{H_{i}^{O}}{\gamma_{0}}\left({\bar{g}}_{i}-{\bar{\gamma }}_{i}\right)$$

Combining expressions defining the geoid-to-quasigeoid separation difference in Equations (14) and (30), the following relation is obtained

(31) $$\Delta \chi_{i,i+1}=\chi_{i+1}-\chi_{i}=NC_{i,i+1}-OC_{i,i+1}$$

Substitution from Equations (30) and (14) yields

(32) $$\frac{H_{i+1}^{O}}{\gamma_{0}}\left({\bar{g}}_{i+1}-{\bar{\gamma }}_{i+1}\right)-\frac{H_{i}^{O}}{\gamma_{0}}\left({\bar{g}}_{i}-{\bar{\gamma }}_{i}\right)=\frac{H_{i}}{\gamma_{0}}\left({\bar{\gamma }}_{i}-{\bar{g}}_{i}\right)-\frac{H_{i+1}}{\gamma_{0}}\left({\bar{\gamma }}_{i+1}-{\bar{g}}_{i+1}\right)$$

To estimate approximation errors caused by using heights H (obtained from levelling measurements) instead of orthometric heights $H^{O}$ in Equation (32), the error analysis is, firstly, written as follows:

(33) $$\begin{array}{l} \varepsilon_{\Delta \chi -NC+OC}=\Delta \chi_{i,i+1}-NC_{i,i+1}+OC_{i,i+1}\\ =\frac{H_{i+1}^{O}}{\gamma_{0}}\left({\bar{g}}_{i+1}-{\bar{\gamma }}_{i+1}\right)-\frac{H_{i}^{O}}{\gamma_{0}}\left({\bar{g}}_{i}-{\bar{\gamma }}_{i}\right)-\frac{H_{i}}{\gamma_{0}}\left({\bar{\gamma }}_{i}-{\bar{g}}_{i}\right)+\frac{H_{i+1}}{\gamma_{0}}\left({\bar{\gamma }}_{i+1}-{\bar{g}}_{i+1}\right)\\ =\frac{H_{i+1}^{O}-H_{i+1}}{\gamma_{0}}\left({\bar{g}}_{i+1}-{\bar{\gamma }}_{i+1}\right)-\frac{H_{i}^{O}-H_{i}}{\gamma_{0}}\left({\bar{g}}_{i}-{\bar{\gamma }}_{i}\right) \end{array}$$

where

(34) $$H_{i+1}^{O}=H_{i}+\Delta H_{i,i+1}+OC_{i,i+1}$$

(35) $$H_{i}^{O}=H_{i+1}-\Delta H_{i,i+1}+OC_{i,i+1}$$

Substitution from Equations (34) and (35) back to Equation (33) then yields

(36) $$\varepsilon_{\Delta \chi -NC+OC}=\frac{H_{i}+\Delta H_{i,i+1}+OC_{i,i+1}-H_{i+1}}{\gamma_{0}}\left({\bar{g}}_{i+1}-{\bar{\gamma }}_{i+1}\right)\;-\frac{H_{i+1}-\Delta H_{i,i+1}+OC_{i,i+1}-H_{i}}{\gamma_{0}}\left({\bar{g}}_{i}-{\bar{\gamma }}_{i}\right)\;=\frac{\Delta H_{i,i+1}-\Delta H_{i,i+1}+OC_{i,i+1}}{\gamma_{0}}\left({\bar{g}}_{i+1}-{\bar{\gamma }}_{i+1}\right)\;-\frac{\Delta H_{i,i+1}-\Delta H_{i,i+1}+OC_{i,i+1}}{\gamma_{0}}\left({\bar{g}}_{i}-{\bar{\gamma }}_{i}\right)\;=\frac{OC_{i,i+1}}{\gamma_{0}}\left({\bar{g}}_{i+1}-{\bar{\gamma }}_{i+1}\right)-\frac{OC_{i,i+1}}{\gamma_{0}}\left({\bar{g}}_{i}-{\bar{\gamma }}_{i}\right)\;=\frac{OC_{i,i+1}}{\gamma_{0}}\left({\bar{g}}_{i+1}-{\bar{g}}_{i}-{\bar{\gamma }}_{i+1}+{\bar{\gamma }}_{i}\right)$$

The error $\varepsilon_{\Delta \chi -NC+OC}$ depends mainly on differences between the mean actual and normal gravity values at levelling benchmarks. Considering even very large differences $\bar{g}-\bar{\gamma }$ in Equation (36) of the order of several hundreds of milligals, the error $\varepsilon_{\Delta \chi -NC+OC}$ is completely negligible (less than 0.1 mm). Consequently, it could be concluded that rigorous definitions of the geoid-to-quasigeoid separation difference according to formulas given in Equations (14) and (30) are equivalent. 

Finally, the approximate definition of the geoid-to-quasigeoid separation in Equation (29) is used to introduce the approximate definition of the geoid-to-quasigeoid separation difference $\Delta {\chi^{\prime }}_{i,i+1}$ in the following form

(37) $$\Delta {\chi^{\prime }}_{i,i+1}={\chi^{\prime }}_{i+1}-{\chi^{\prime }}_{i}=\frac{H_{i+1}^{N}}{\gamma_{0}}\Delta g_{i+1}^{\mathrm{SPB}}-\frac{H_{i}^{N}}{\gamma_{0}}\Delta g_{i}^{\mathrm{SPB}}$$

Again, normal heights in Equation (37) can be disregarded. Instead, heights H obtained from levelled height differences can be used. Such an assumption introduces errors in values of $\chi^{\prime }$ typically less than ±1 mm that correspond to even smaller errors in values of $\Delta {\chi^{\prime }}_{i,i+1}$ so that

(38) $$\Delta {\chi^{\prime }}_{i,i+1}≅\frac{H_{i+1}^{}}{\gamma_{0}}\Delta g_{i+1}^{\mathrm{SPB}}-\frac{H_{i}^{}}{\gamma_{0}}\Delta g_{i}^{\mathrm{SPB}}$$

Consequently,

(39) $$NC_{i,i+1}-OC_{i,i+1}≅\Delta {\chi^{\prime }}_{i,i+1}=\frac{H_{i+1}^{}}{\gamma_{0}}\Delta g_{i+1}^{\mathrm{SPB}}-\frac{H_{i}^{}}{\gamma_{0}}\Delta g_{i}^{\mathrm{SPB}}$$

Rigorous and approximate relations between the normal and orthometric correction difference and the geoid-to-quasigeoid separation difference were derived and presented above. The rigorous definition in Equation (30) was described by means of the difference between the actual mean and normal gravity values. The corresponding approximate relation in Equation (39) is described as a function of the simple planar Bouguer gravity anomaly values. If the Poincaré-Prey gravity gradient closely approximates the actual vertical gravity gradient inside the topography, both definitions should provide results that differ less than ±1 mm. In other words, the relation $NC_{i,i+1}-OC_{i,i+1}≅\Delta {\chi^{\prime }}_{i,i+1}$ in Equation (39) should be accurate enough to be applicable for a practical realization of Helmert orthometric heights ${\tilde{H}}^{O}$ and a conversion between the Helmert orthometric and Molodensky normal heights (i.e., $\chi^{\prime }$) by using the formula in Equation (29). This theoretical aspect is numerically investigated next. 

## 3. Numerical Procedures

The accuracy of the geoid-to-quasigeoid separation differences was assessed at a vertical geodetic control in Hong Kong, practically realized by the Vertical Control Network 2022 (VCN2022). Since gravity values along levelling lines were not measured directly, detailed terrestrial and marine gravity measurements were used to interpolate gravity values along levelling lines [55]. Interpolated gravity values (at levelling benchmarks) were then used to compute the orthometric and normal corrections to measured levelling height differences, and the entire levelling network was readjusted. The newly determined normal and orthometric heights of levelling benchmarks were presented as the VCN2022 solution. The adjustment of the orthometric and normal levelling networks of the VCN2022 attained a −0.2 and 2.0 mm misclosure, respectively. 

**Figure 2:**
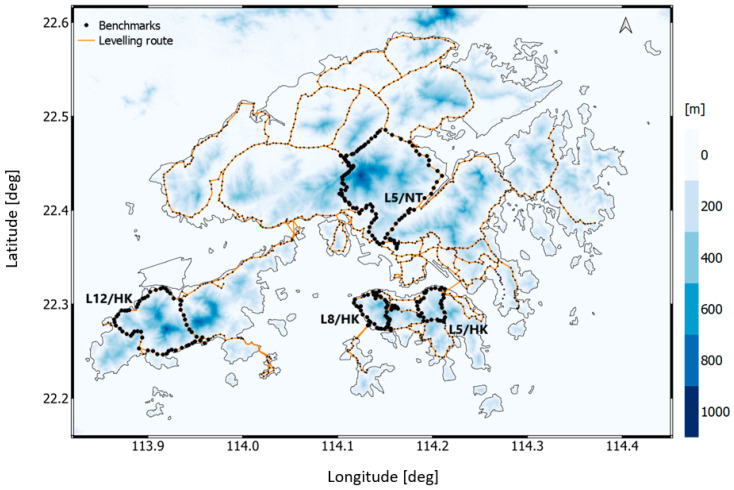
The VCN2022 levelling network. The L5/NT, L5/HK, L8/HK, and L12/HK denote selected four closed levelling loops used in the numerical study. Topographic elevations are extracted from the 1 arc second SRTM DEM and are in blue.

To keep the presentation simple but still instructive, we conducted the numerical analysis only along four closed levelling loops of the VCN2022 levelling network which were characterized by the largest topographic elevation changes in Hong Kong. The location of the selected VCN2022 levelling sections is illustrated in Figure 2.

### 3.1. Gravity Data Interpolation

Since gravity data from mainland China are not publicly available, [55] applied a simple gravity data interpolation instead of more refined methods based on computing the complete spherical Bouguer gravity anomalies [56] and their downward continuation (by solving the inverse of the Poisson integral equation). First, they used the measured free-air gravity anomalies $\Delta g^{\mathrm{FA}}$ to compute the simple planar Bouguer anomalies $\Delta g^{\mathrm{SPB}}$ by applying the Bouguer gravity reduction. They then used the simple planar Bouguer gravity anomalies (at gravity sites) to interpolate the corresponding values at levelling benchmarks by applying the inverse distance weighted mean. This method was selected based on testing a performance of various interpolation techniques, particularly by applying the kriging, natural neighbor, least-squares collocation, nearest neighbor, and radial basis functions for the gravity data interpolation. According to their results, maximum differences in interpolated gravity values (i.e., the simple planar Bouguer gravity anomalies) from these methods at levelling benchmarks were within ±5 mGal. Such gravity differences correspond to differences in computed values of the normal and orthometric corrections less than ±1 mm (cf. [55]). Finally, they converted the interpolated simple planar Bouguer gravity anomalies to the free-air gravity anomalies at levelling benchmarks. A gravity data interpolation by applying only the Bouguer gravity reduction (while disregarding the terrain gravity correction) is obviously less accurate. A discussion of this aspect is postponed until Section 5. Nevertheless, it is worth already clarifying here that this factor does not affect the numerical findings in this study. 

### 3.2. Orthometric and Normal Heights 

Nsiah Ababio and Tenzer [55] used the free-air gravity anomalies $\Delta g^{\mathrm{FA}}$ at levelling benchmarks to compute the orthometric and normal corrections to measured levelling height differences. For 2 consecutive levelling benchmarks *i* and *i* + 1 of a levelling segment, the orthometric correction $OC_{i,i+1}$ was computed according to [25] using the following equation:

(40) $$OC_{i,i+1}=\frac{1}{{\bar{g}}_{i+1}}\left(\frac{\Delta g_{i}^{\mathrm{FA}}+\Delta g_{i+1}^{\mathrm{FA}}}{2}+\frac{\partial \gamma }{\partial h}\frac{H_{i}+H_{i+1}}{2}+\frac{\gamma_{0,}{}_{i}+\gamma_{0,i+1}}{2}-{\bar{g}}_{i+1}\right)\Delta h_{i,i+1}+H_{i}^{}\left(\frac{{\bar{g}}_{i}}{{\bar{g}}_{i+1}}-1\right)$$

By analogy with Equation (40), the normal correction $NC_{i,i+1}$ was computed from

(41) $$NC_{i,i+1}=\frac{1}{{\bar{\gamma }}_{i+1}}\left(\frac{\Delta g_{i}^{\mathrm{FA}}+\Delta g_{i+1}^{\mathrm{FA}}}{2}+\frac{\partial \gamma }{\partial h}\frac{H_{i}+H_{i+1}}{2}+\frac{\gamma_{0,}{}_{i}+\gamma_{0,i+1}}{2}-{\bar{\gamma }}_{i+1}\right)\Delta h_{i,i+1}+H_{i}^{}\left(\frac{{\bar{\gamma }}_{i}}{{\bar{\gamma }}_{i+1}}-1\right)$$

Adopting Helmert’s definition of orthometric heights, the mean gravity in Equation (40) was computed from

(42) $${\bar{g}}_{i}=g_{i}+2\;\pi \;G\;\rho^{T}H_{i}$$

where the surface gravity $g_{i}$ was computed from the free-air gravity anomaly $\Delta g^{\mathrm{FA}}$ as follows:

(43) $$g_{i}=\Delta g_{i}^{\mathrm{FA}}+\frac{\partial \gamma }{\partial h}H_{i}+\gamma_{0,}{}_{i}$$

Substitution from Equation (43) to Equation (42) yields

(44) $${\bar{g}}_{i}=\Delta g_{i}^{FA}+\frac{\partial \gamma }{\partial h}H_{i}+\gamma_{0,}{}_{i}+2\;\pi \;G\;\rho^{T}H_{i}$$

Equations (21) and (44) are equivalent. The last term on the right-hand side of Equation (44) is computed for the topographic density value of 2670 kg m^−3^. To check the correctness of both results, the orthometric and normal corrections were also computed according to Equations (5) and (6), finding that both results are equal. 

The orthometric height differences $\Delta H_{i,i+1}^{O}$ and the normal height differences $\Delta H_{i,i+1}^{N}$ between levelling benchmarks *i* and *i* + 1 were computed according to Equations (10) and (11) by applying the orthometric $OC_{i,i+1}$ and normal $NC_{i,i+1}$ corrections to measured levelling height differences $\Delta H_{i,i+1}$, respectively. The newly determined orthometric $\Delta H_{i,i+1}^{O}$ and normal $\Delta H_{i,i+1}^{N}$ height differences were then used to readjust the entire levelling network and to compute the normal and orthometric heights of levelling benchmarks.

### 3.3. The Orthometric and Normal Correction Differences and the Geoid-to-Quasigeoid Separation Differences 

Values of the orthometric corrections $OC_{i,i+1}$ and the normal corrections $NC_{i,i+1}$ prepared by [55] were used to compute the orthometric and normal correction differences $OC_{i,i+1}-NC_{i,i+1}$ along four selected closed levelling loops. The geoid-to-quasigeoid separation differences $\Delta {\chi^{\prime }}_{i,i+1}={\chi^{\prime }}_{i+1}-{\chi^{\prime }}_{i}$ were computed according to Equation (39) from the Bouguer gravity anomaly values $\Delta g_{i+1}^{\mathrm{SPB}}$ and $\Delta g_{i}^{\mathrm{SPB}}$. For completeness, the geoid-to-quasigeoid separation values ${\chi^{\prime }}_{i}$ were computed according to Equation (29) and compared with the differences between the Molodensky normal heights and the Helmert orthometric heights at levelling benchmarks (obtained after the adjustment of the whole levelling network). 

## 4. Results

**Figure 3:**
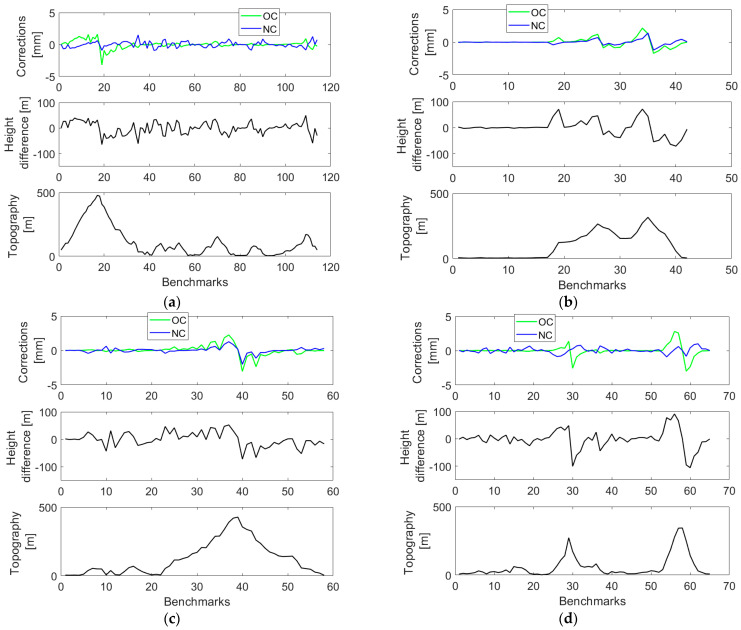
Values of the orthometric and normal corrections (upper panels) along the VCN2022 levelling profiles: (**a**) L5/NT, (**b**) L5/HK (**c**) L8/HK, and (**d**) L12/HK. Height differences of individual segments between levelling benchmarks and the topographic relief along levelling profiles are plotted in middle and lower panels, respectively.

**Figure 4:**
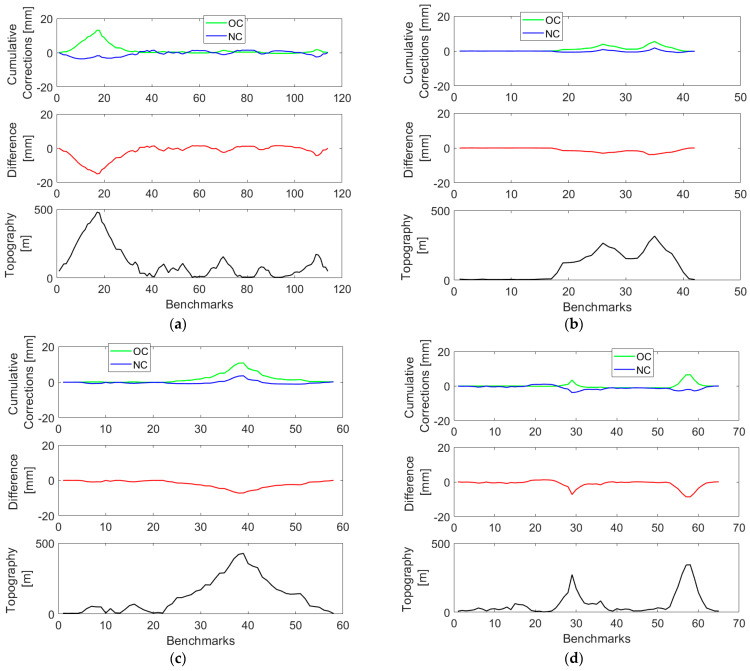
Cumulative values of the orthometric and normal corrections (upper panels) along the VCN2022 levelling profiles: (**a**) L5/NT, (**b**) L5/HK (**c**) L8/HK, and (**d**) L12/HK, and their differences (middle panels). The topographic relief along levelling profiles is plotted in lower panels.

The orthometric and normal corrections and their cumulative values along four selected closed levelling loops are plotted in Figure 3 and Figure 4 (with statistical summaries in Table 1 and Table 2), respectively, where the topographic relief and height differences (between individual levelling segments) are also shown. Values of the orthometric correction are mostly within ±3 mm, and values of the normal correction vary largely within ±2 mm (Table 1). 

Both corrections reach maximum (absolute) values at levelling sections characterized by the largest elevation changes. Cumulative values of the orthometric correction closely mimic a topographic relief, while this trend in cumulative values of the normal correction is less pronounced (see Figure 4, upper panels) so that their differences (see Figure 4, middle panels) are mostly attributed to the cumulative orthometric correction. 

**Figure 5:**
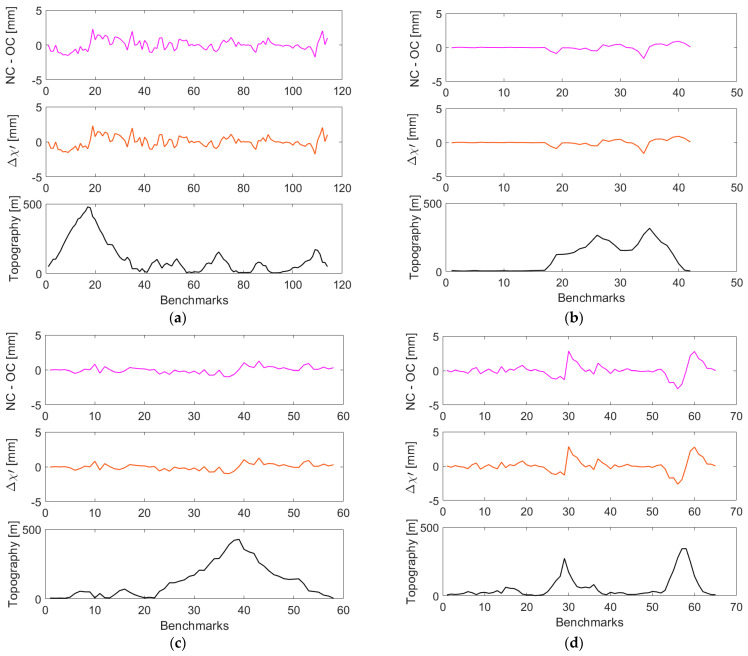
Comparison of the orthometric and normal correction differences (upper panels) with the geoid-to-quasigeoid separation differences (middle panels) along the VCN2022 levelling profiles: (**a**) L5/NT, (**b**) L5/HK (**c**) L8/HK, and (**d**) L12/HK; values of the orthometric and normal correction differences (upper panels) and values of the geoid-to-quasigeoid separation differences (middle panels). The topographic relief along levelling profiles is plotted in lower panels.

As discussed in Section 2, the orthometric and normal correction differences should be equal (or very similar) with the geoid-to-quasigeoid separation differences computed for individual levelling segments. This aspect was inspected in Figure 5 (with the statistical summary in Table 3), where values of the orthometric and normal correction differences were plotted and compared with values of the geoid-to-quasigeoid separation differences $\Delta {\chi^{\prime }}_{i,i+1}$. As can be seen, the geoid-to-quasigeoid separation differences fully agree with the orthometric and normal correction differences. 

**Figure 6:**
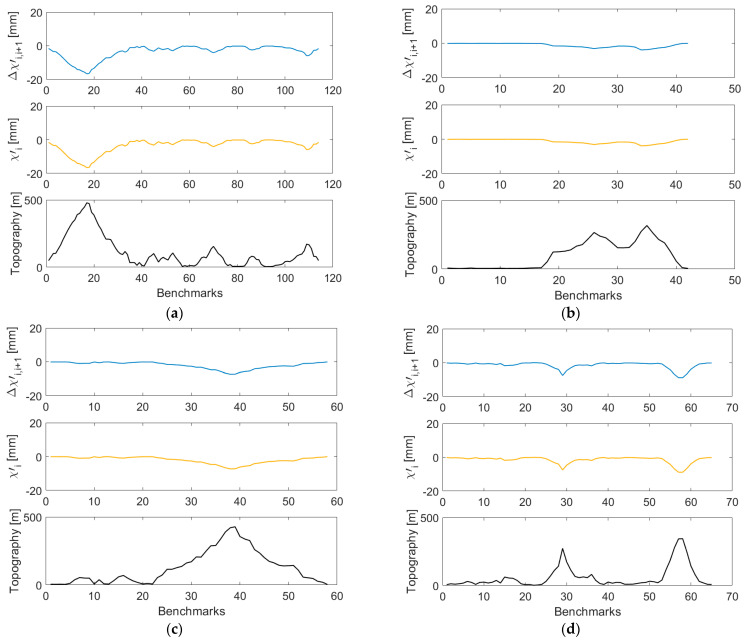
Comparison of the geoid-to-quasigeoid separation computed pointwise at levelling benchmarks (upper panels) with the corresponding values (at levelling benchmarks) computed cumulatively from the geoid-to-quasigeoid separation differences (middle panels) along the VCN2022 levelling profiles: (**a**) L5/NT, (**b**) L5/HK (**c**) L8/HK, and (**d**) L12/HK. The topographic relief along levelling profiles is plotted in lower panels.

For completeness, we compared values of the geoid-to-quasigeoid separation ${\chi^{\prime }}_{i}$ at levelling benchmarks with corresponding values computed cumulatively from the geoid-to-quasigeoid separation differences $\Delta {\chi^{\prime }}_{i,i+1}$. The results are plotted in Figure 6 (with the statistical summary in Table 4). 

**Figure 7:**
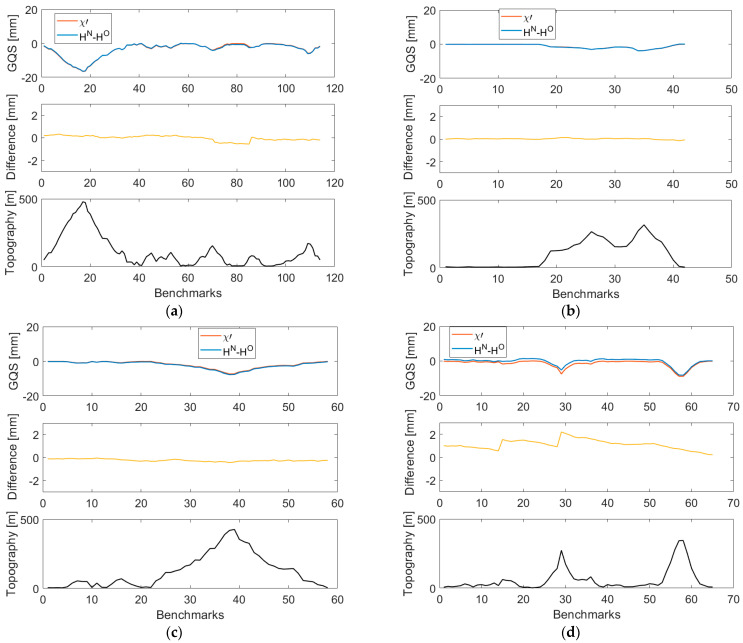
The geoid-to-quasigeoid separation and the differences between the Molodensky normal heights and the Helmert orthometric heights (upper panels) and their differences (middle panels) at levelling benchmarks along the VCN2022 levelling profiles: (**a**) L5/NT, (**b**) L5/HK (**c**) L8/HK, and (**d**) L12/HK. The topographic relief along levelling profiles is plotted in lower panels.

As seen, cumulative and pointwise values (at levelling benchmarks) agree. Finally, we compared values of the geoid-to-quasigeoid separation ${\chi^{\prime }}_{i}$ with the differences between the Molodensky normal heights and the Helmert orthometric heights at levelling benchmarks obtained after the readjustment of the whole network. The results are plotted in Figure 7, with the statistical summary of the results in Table 5. As seen, values of the geoid-to-quasigeoid separation differ from differences between the Molodensky normal heights and the Helmert orthometric heights. The largest differences, exceeding even 2 mm, are seen along the L12/HK levelling loop in the Lantau Island. The existence of these relatively large differences is discussed in the next section. 

## 5. Discussion

In Hemert’s definition of orthometric heights, the Poincaré-Prey gravity gradient was adopted to approximate the actual gravity gradient. The mean gravity within the topography was then approximated by the surface gravity continuing downward to a midpoint by using the normal gravity gradient and by applying the Poisson equation to take the topography into consideration. To convert the Molodensky normal heights to the Helmert orthometric heights and vice versa, the geoid-to-quasigeoid separation was defined as a function of the simple planar Bouguer gravity anomaly $\Delta g^{\mathrm{SPB}}$ based on adopting approximations equivalent to those used in Helmert’s definition of orthometric heights and further rearranging the expression in terms of $\bar{g}-\bar{\gamma }$ to its final form described as a function of $\Delta g^{\mathrm{SPB}}$. It is thus expected that the expressions for computing the normal and orthometric correction differences and the geoid-to-quasigeoid separation differences provide the same (or very similar) results. 

As demonstrated in the numerical examples, this assumption is correct. Both computed differences are almost the same with differences reaching less than ±0.1 mm. It is worth noting that [55] inspected the reliability of intermediate numerical steps involved to compute the normal and orthometric correction differences and the geoid-to-quasigeoid separation differences, assuring that findings presented in this study are valid. Particularly, they demonstrated that the application of different gravity interpolation techniques does not affect the accuracy. Obviously, the selection of a gravity interpolation technique in mountainous regions with much higher elevation changes requires a careful analysis. 

These findings ascertain that the computation of the geoid-to-quasigeoid separation from the Bouguer gravity data is fully compatible with Helmert’s definition of orthometric heights. The expression for computing the geoid-to-quasigeoid separation from the simple planar Bouguer gravity anomaly provides the result that is equal to the geoid-to-quasigeoid separation differences.

Finally, we demonstrated that the geoid-to-quasigeoid separation differs from differences between the Molodensky normal and Helmert orthometric heights obtained after the levelling network readjustment (carried out individually for the normal and orthometric height differences), particularly along the L12/HK closed levelling loop. According to our results, the differences between the geoid-to-quasigeoid separation and differences between the Molodensky normal and Helmert orthometric heights there exceed even 2 mm. This inconsistency is explained mainly by the propagation of errors in measured levelling height differences. This was confirmed by the analysis of the adjusted levelled height differences. The results of this analysis revealed that levelled height differences along the L12/HK closed levelling loop located in the Lantau Island are systematically affected by errors of levelling measurements conducted along the bridge, which connect the island with the rest of the territories. Elsewhere, these differences are much smaller. 

## 6. Summary and Concluding Remarks

We have demonstrated that the geoid-to-quasigeoid separation defined as a function of the simple planar Bouguer gravity anomaly is fully compatible with Helmert’s definition of orthometric heights. Since both definitions involve the same assumptions regarding the approximation of the actual gravity gradient by the Poincaré-Prey gravity reduction, the computation of the geoid-to-quasigeoid separation in terms of the simple planar Bouguer gravity anomaly in Equation (29) introduces errors that should not exceed more than ±1 mm (except for extremely large topographic elevations particularly in the Himalayas, Tibet, and the Andes). Obviously, the mean gravity values computed by means of applying the Poincaré-Prey gravity reduction might still be quite inaccurate. Nevertheless, the approximately-computed geoid-to-quasigeoid separation (from the Bouguer gravity data) is consistent with the Helmert’s definition of orthometric heights that has been, until now, exclusively used for a practical realization of geodetic vertical controls in countries where orthometric heights are officially adopted. 

## Figures and Tables

**Table 1:** Statistics of values of the orthometric and normal corrections and the topographic heights and height differences of individual segments between benchmarks along the VCN2022 levelling profiles for the selected loops L5/NT, L5/HK, L8/HK, and L12/HK.

Orthometric Corrections				
LOOPS	MIN [mm]	MAX [mm]	MEAN [mm]	STD [mm]
L5/NT	−3.2	1.6	0.0	0.6
L5/HK	−1.7	2.2	0.0	0.7
L8/HK	−3.1	2.2	0.0	0.8
L12/HK	−3.0	2.8	0.0	0.8
Normal Corrections				
L5/NT	−0.9	1.5	0.0	0.4
L5/HK	−1.2	1.4	0.0	0.4
L8/HK	−2.0	1.3	0.0	0.4
L12/HK	−0.9	1.0	0.0	0.4
Height differences				
LOOPS	MIN [m]	MAX [m]	MEAN [m]	STD [m]
L5/NT	−64.565	49.667	−0.036	23.642
L5/HK	−70.247	71.207	0.000	31.656
L8/HK	−71.900	52.568	0.000	26.868
L12/HK	−105.580	90.796	−1.829	35.269
Topography				
L5/NT	4.109	478.233	110.541	118.707
L5/HK	3.850	316.242	102.053	100.755
L8/HK	3.844	427.394	125.350	121.717
L12/HK	2.150	344.152	60.358	80.841

**Table 2:** Statistics of cumulative orthometric and normal corrections and their differences.

Cumulative Orthometric Correction				
LOOPS	MIN [mm]	MAX [mm]	MEAN [mm]	STD [mm]
L5/NT	−0.4	13.2	1.5	3.0
L5/HK	−0.1	5.5	1.1	1.4
L8/HK	−0.1	10.8	1.8	2.8
L12/HK	−1.0	6.6	0.1	1.5
Cumulative normal correction				
L5/NT	−3.6	1.5	−0.5	1.5
L5/HK	−0.8	1.8	−0.1	0.5
L8/HK	−1.1	3.6	−0.2	1.0
L12/HK	−3.8	1.1	−1.0	1.1
Differences in cumulative corrections				
L5/NT	−15.0	1.5	−2.1	4.2
L5/HK	−3.8	0.0	−1.2	1.2
L8/HK	−7.2	0.0	−2.0	2.1
L12/HK	−8.6	1.2	−1.1	2.2

**Table 3:** Statistics of values of the orthometric and normal correction differences and cumulative values of the geoid-to-quasigeoid separation differences.

Normal and Orthometric Correction Differences (NC–OC)				
LOOPS	MIN [mm]	MAX [mm]	MEAN [mm]	STD [mm]
L5/NT	−1.8	2.3	0.0	0.8
L5/HK	−1.6	0.9	0.0	0.4
L8/HK	−1.0	1.3	0.0	0.5
L12/HK	−2.6	2.8	0.1	0.9
Geoid-to-quasigeoid separation differences				
L5/NT	−1.8	2.3	0.0	0.8
L5/HK	−1.6	0.9	0.0	0.4
L8/HK	−1.0	1.3	0.0	0.5
L12/HK	−2.6	2.8	0.1	0.9

**Table 4:** Statistics of values of the geoid-to-quasigeoid separation computed pointwise at levelling benchmarks and the corresponding values (at levelling benchmarks) computed cumulatively from the geoid-to-quasigeoid separation differences.

Cumulatively Computed Values of the Geoid-to-Quasigeoid Separation				
LOOPS	MIN [mm]	MAX [mm]	MEAN [mm]	STD [mm]
L5/NT	−16.6	−0.1	−3.7	4.2
L5/HK	−3.9	−0.1	−1.2	1.2
L8/HK	−7.3	−0.1	−2.1	2.1
L12/HK	−8.9	−0.1	−1.6	2.1
Pointwise computed values of the geoid-to-quasigeoid separation				
L5/NT	−16.6	−0.1	−3.7	4.2
L5/HK	−3.9	−0.1	−1.2	1.2
L8/HK	−7.3	−0.1	−2.1	2.1
L12/HK	−8.9	−0.1	−1.6	2.1

**Table 5:** Statistics of values of the geoid-to-quasigeoid separation (computed pointwise) and differences between the Molodensky normal heights and the Helmert orthometric heights at levelling benchmarks.

Pointwise Geoid-to-Quasigeoid Separation				
LOOPS	MIN [mm]	MAX [mm]	MEAN [mm]	STD [mm]
L5/NT	−16.615	−0.122	−3.667	4.231
L5/HK	−3.881	−0.054	−1.239	1.220
L8/HK	−7.272	−0.069	−2.050	2.058
L12/HK	−8.857	−0.060	−1.571	2.105
Differences between Molodensky normal heights and Helmert orthometric heights				
L5/NT	−16.500	0.000	−3.689	4.146
L5/HK	−3.900	0.000	−1.257	1.230
L8/HK	−7.700	−0.200	−2.279	2.123
L12/HK	−8.200	1.300	−0.466	2.149
Differences				
L5/NT	−0.552	0.326	−0.020	0.227
L5/HK	−0.142	0.144	0.018	0.053
L8/HK	−0.428	−0.034	−0.230	0.096
L12/HK	0.226	2.196	1.105	0.422

## Data Availability

The data that support the findings of this study are available on request from the corresponding author.
